# Transforming the evaluation of agrochemicals

**DOI:** 10.1002/ps.7148

**Published:** 2022-09-14

**Authors:** Douglas C Wolf, Yadvinder Bhuller, Rhian Cope, Marco Corvaro, Richard A Currie, John Doe, Adriana Doi, Gina Hilton, Jyotigna Mehta, David Saltmiras, Fiona Sewell, Maria Trainer, Sandrine E Déglin

**Affiliations:** ^1^ Product Safety Syngenta Crop Protection Greensboro NC USA; ^2^ Executive Director's Office, Pest Management Regulatory Agency Health Canada Ottawa ON Canada; ^3^ Australian Pesticides and Veterinary Medicines Authority Armidale NSW Australia; ^4^ Regulatory Toxicology, Human Safety Corteva Agriscience Rome Italy; ^5^ Product Safety Early Stage Research Syngenta Crop Protection Jealotts Hill United Kingdom; ^6^ Pharmacy and Biomolecular Sciences Liverpool John Moores University Liverpool UK; ^7^ Regulatory Science Crop Protection BASF Crop Protection Research Triangle Park NC USA; ^8^ PETA Science Consortium International e.v Stuttgart Germany; ^9^ ADAMA Agricultural Solutions Ltd Reading UK; ^10^ Human Safety Bayer CropScience Chesterfield MO USA; ^11^ Toxicology and Regulatory Sciences National Centre for the Replacement Refinement and Reduction of Animals in Research (NC3Rs) London UK; ^12^ Health and Environmental Sciences Institute Washington DC USA

**Keywords:** safety assessment, pesticide, testing, crop protection, risk assessment

## Abstract

The present agrochemical safety evaluation paradigm is long‐standing and anchored in well‐established testing and evaluation procedures. However, it does not meet the present‐day challenges of rapidly growing populations, food insecurity, and pressures from climate change. To transform the current framework and apply modern evaluation strategies that better support sustainable agriculture, the Health and Environmental Sciences Institute (HESI) assembled a technical committee to reframe the safety evaluation of crop‐protection products. The committee is composed of international experts from regulatory agencies, academia, industry and nongovernmental organizations. Their mission is to establish a framework that supports the development of fit‐for‐purpose agrochemical safety evaluation that is applicable to changing global, as well as local needs and regulatory decisions, and incorporates relevant evolving science. This will be accomplished through the integration of state‐of‐the‐art scientific methods, technologies and data sources, to inform safety and risk decisions, and adapt them to evolving local and global needs. The project team will use a systems‐thinking approach to develop the tools that will implement a problem formulation and exposure driven approach to create sustainable, safe and effective crop protection products, and reduce, replace and refine animal studies with fit‐for‐purpose assays. A new approach necessarily will integrate the most modern tools and latest advances in chemical testing methods to guarantee the robust human and environmental safety and risk assessment of agrochemicals. This article summarizes the challenges associated with the modernization of agrochemical safety evaluation, proposes a potential roadmap, and seeks input and engagement from the broader community to advance this effort. © 2022 Health and Environmental Sciences Institute (HESI). *Pest Management Science* published by John Wiley & Sons Ltd on behalf of Society of Chemical Industry.

## Graphical abstract

This article presents the needs for a new crop‐protection product evaluation paradigm that is fit‐for‐purpose, integrative of the best available science, and adapted to global and local needs. It proposes a roadmap to help achieve that unique vision, and calls for input and engagement from all stakeholders.

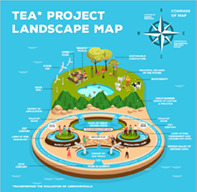

## 1 INTRODUCTION

Many changes in the regulatory landscape and associated scientific advancements have taken place since a series of papers were published, in the early 2000s, to detail a tiered, scientifically appropriate, strategy to evaluate the potential human health risks of agrochemicals.^1^, ^2^, ^3^, ^4^ These publications illustrated how the proposed approach would increase the increased efficiency in the risk assessment process, and reduced animal and resource use. Since their publication, there has been some progress with the elimination of the routine requirement for the one‐year dog study, increased use of toxicokinetics and mechanistic data, new guidelines of improved reproduction studies, and implementation of tiered testing strategies. In addition, there has been widespread incorporation of new approach methodologies (NAMs) with greater relevance for human safety characterization.^5^ The rate and magnitude of scientific advancement over the past several decades provides the opportunity to modernize the approach to safety assessment of agrochemicals. Although the previous HESI effort addressed only human health risks, the present initiative will address both human health and environmental safety and risk to identify efficiencies in and integration of associated processes with humans as part of – and not separate from – the environment (www.onehealthinitiative.com; www.cdc.gov/onehealth).^6^, ^7^

Success in modifying the need for animal‐based toxicity studies for agrochemical evaluation is illustrated by the U.S. Environmental Protection Agency's (US EPA's) waiver program. This program shows the potential and emphasizes the importance of engagement and dialogue between regulatory agencies and the regulated community.^8^, ^9^, ^10^ To achieve the desired change, engagement of all stakeholders including government regulatory scientists and risk managers, academic scientists, representatives from relevant nongovernmental organizations, and industry scientists is necessary to establish relevant and useful decision‐making processes that are protective of public health and the environment.^10^, ^11^

## 2 MOVING BEYOND ONE‐TO‐ONE REPLACEMENT STRATEGIES

Although the existing agrochemical safety evaluation paradigm is well‐established and anchored in trusted internationally accepted test guidelines there is a need to reevaluate this work through a broader, systems‐thinking lens.^12^ The systems approach highlights connections and encourages a shift of mindset to keep up with new challenges and the ever‐expanding global agricultural needs. Advancing innovation rapidly and efficiently within the agrochemical community will require a coordinated effort involving all stakeholders and leaders, and collecting their input not only to ensure that scientific and technical needs are addressed, but also to encourage uniform testing requirements and uniform data evaluation, and ultimately support the safe use of agrochemicals. This effort will transcend the concept of mutual acceptance of data by creating a globally accepted framework for knowledge generation and evaluation that informs safety based on relevant risk assessments.

Agriculturalists are faced with numerous challenges including climate change, invasive species, producing more food with less land and water, and resistance of pests to available control methods. Increased need for innovative tools coupled with integrated pest management (IPM) strategies are necessary to meet these challenges. Lack of resiliency in the global food production system (https://www.fao.org/publications/sofi/2021/en/) and the expanding use of agricultural products to other sector such as energy production and botanical pharmaceuticals has dramatically increased the need for safe and effective plant protection products.

Numerous collaborations have led to the development of NAMs and frameworks now available to inform human and environmental safety and risk. Notable successes include the US EPA's Computational Toxicology Program (https://comptox.epa.gov/dashboard/), HESI's RISK21 framework (www.risk21.org), the OECD Guidelines, and Integrated Approaches to Testing and Assessment developed by the Organization for Economic Cooperation and Development (OECD IATAs; https://www.oecd.org/chemicalsafety/risk-assessment/iata-integrated-approaches-to-testing-and-assessment.htm), and various Tox21 assays (https://tox21.gov/). In qualifying NAMs, it is critical to make sure that they are supported by information that accurately evaluates agrochemicals and their formulations.^13^ Large international consortia, the OECD and major government institutions, have developed fit‐for‐purpose NAMs and created international and country‐specific guidelines for chemical evaluation. These efforts repeatedly have shown how alternative approaches can be used in lieu of traditional whole animal tests.^10^, ^11^, ^14^, ^15^, ^16^, ^17^, ^18^ Although there have been initiatives and government directives to set the stage for what the future of agrochemical safety testing might look like, no concerted effort has addressed how to implement NAMs holistically rather than in a one‐to‐one replacement for the traditional OECD Test Guidelines. Such an approach requires a comprehensive way to evaluate human and environmental risks from agrochemicals to improve the overall safety assessment framework while integrating these alternative methods, and is addressed in this HESI project.

## 3 CREATING A NEXT‐GENERATION FRAMEWORK FOR AGROCHEMICALS

**Figure 1:**
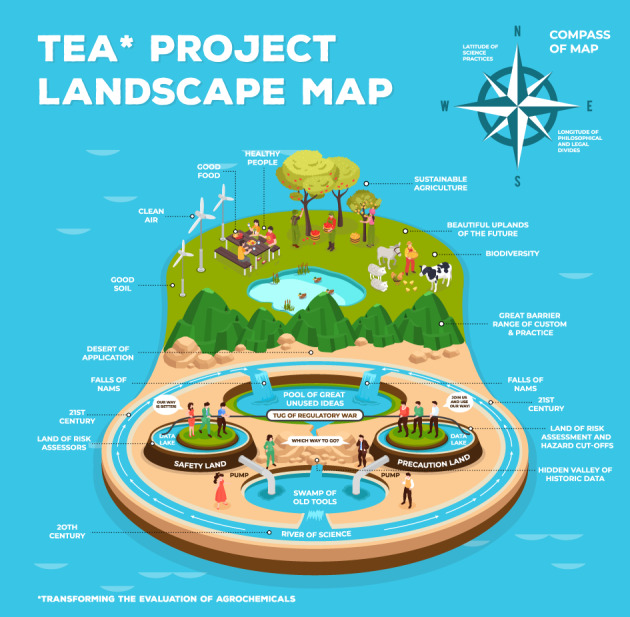
Techno‐regulatory conceptual landscape map illustrating the various parts that need to be considered in a conceptual model. A clear common goal is to create an agricultural system that provides a just, sustainable and healthy environment. Over time, continuous investment has resulted in scientific developments through the 20th and 21st centuries (the ‘river of science’) that has produced and will continue to produce substantial scientific progress in the development of NAMs. Although existing tools have generated useful information, often the associated data are hidden in inaccessible documents in repositories that prevent their effective use. Additionally, many of these old tools increasingly are seen as unethical, unfit or impractical for use in contemporary safety evaluation. To date, apart from a few notable exceptions, most of the NAM advances remain unused in regulatory application. This pool of currently unused ideas and the currently inaccessible historic toxicology data can become the new toolkit that will significantly modernize agrochemical safety assessments – but only if we can overcome the many various technical and conceptual barriers to their application. We also must acknowledge that there are philosophical differences between countries in terms of their use of solely science‐based risk assessments (‘safety land’) and those regions that also have additional emphasis on hazard‐based precautionary criteria in addition to their safety assessments (‘precaution land’). This philosophical difference naturally means that there is not one approach but an acknowledgement that different routes from these different starting points in our techno‐regulatory landscape will have to be developed, so that progress towards a common goal – albeit at different speeds and by different routes – will traverse the existing barriers. Therefore, the TEA project aims to map this current landscape in detail to inform building the roadmaps through the existing and anticipated future scientific, technical, regulatory, policy and legal landscapes that will enable a transformation of the evaluation of crop protection methods, and better reflect current and emerging evidence‐based requirements for agrochemicals.

In order to develop a framework assessing the human and environmental safety of new agrochemical plant protection products, HESI created a project named ‘Transforming the Evaluation of Agrochemicals (TEA; https://hesiglobal.org/transforming-the-evaluation-of-agrochemicals-tea/).’The TEA project is attempting to create an evidence‐based roadmap that provides direction for transforming the evaluation of agrochemicals based on current and emerging science about human and environmental safety (Fig. 1). It also is exploring the current scientific, technical, regulatory, legal and philosophical landscape of agrochemical evaluation in many different regions and countries.

The current animal‐based toxicity testing approach originally was developed in the late 1940s and has not fundamentally changed, but new tests have been added, new assays validated, some studies refined and process adaptations implemented to address ever‐expanding concerns, creating the current set of test guidelines.^19^, ^20^ Many NAMs and new conceptual frameworks for safety assessment have been, and continue to be, developed, and regulatory and legal systems have evolved, to fit the needs of individual countries linked together by requirements to facilitate global trade in agricultural commodities.

The basic approaches to agrochemical evaluation are broadly divided into risk‐based regimes and regimes that incorporate specific hazard‐based criteria into their preliminary decision‐making. The diversity of technical, regulatory and philosophical positions adds a layer of challenge that can only be addressed through the identification and acceptance of opportunities to use new approaches that accelerate desirable innovations in agriculture. The TEA project will map social and techno‐regulatory landscapes and identify potential paths through identified barriers to enable a more rapid and harmonized movement towards a future that sustainably supports agriculture.

A critical first step for any project is to identify and clearly formulate the problems to be solved. This focuses the project's efforts and frames the issues that need to be addressed.^21^ After thinking through the many issues associated with agrochemical assessment, the TEA project team created the following problem statement:

Establish the landscape or map that supports the development of fit‐for‐purpose safety

evaluation of agrochemicals, is applicable to changing global as well as local needs

for regulatory decisions, and can incorporate relevant evolving science inputs.

**Figure 2:**
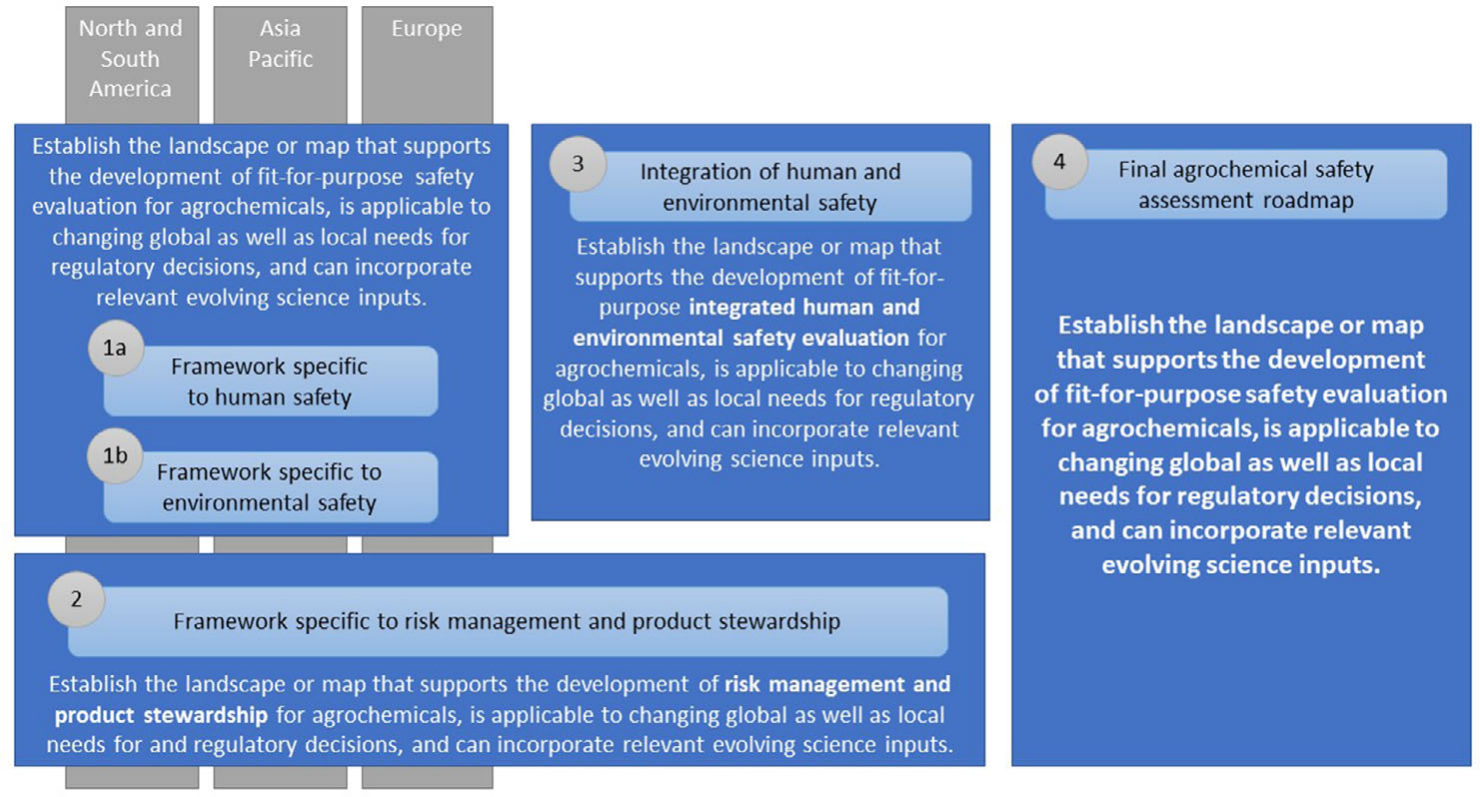
Project structure to address each problem statement. As this is a global activity, separate region‐specific subgroups within each subcommittee were established to maximize input and fully address each problem. The numbering represents the order in which these problem statements will be addressed as they build on each other, for example 1a and 1b are being addressed simultaneously with separate groups of subject matter experts.

The problem statement was further adapted to focus on target areas including human safety, environmental impacts, the integration of human and environmental safety, and the information of risk management decisions and product stewardship needs. The project was then organized into three subcommittees that would explore each of these areas to identify relevant hypotheses and additional issues that should be addressed (Fig. 2).^10^, ^22^

## 4 OPTIMIZING AGROCHEMICAL SAFETY

The innovations implemented in agriculture over the coming decades will impact pesticide use qualitatively and quantitatively. Exposure scenarios are likely to differ markedly from current scenarios associated with traditional agricultural practices. Current evaluation tools, methodologies and regulatory assessment paradigms have varying degrees of flexibility and lag behind emerging scientific questions that must be answered to improve modern agricultural production.

The evolution from problem formulation to a pragmatic and useful framework requires a number of steps if it is going to provide a global strategy (Fig. 2). Evaluation of local societal needs and identification of ways to meet them will occur through adaptations in policy and regulation. The lack of global harmonization has been a persistent barrier to acceptance of new testing and assessment paradigms for agrochemicals.

The incremental change model is the most common approach to transitioning to a new paradigm; however, incremental change can be a lengthy process. For example, the path to achieve a single change in agrochemical safety procedures to end the one‐year dog test was a long journey. Although the first evidence that this test was redundant emerged in the 1980s,^23^ it was recognized by major regulatory authorities much later, in 2006.^24^ Only recently have OECD countries decided to make the one‐year dog test a conditional requirement and necessary only on a case‐by‐case basis.^25^ The rapid progress and advances in the sciences, agrochemical risk assessment and agricultural practice have made gradualism and incrementalism to develop internationally accepted testing and assessment methods ineffective. A paradigm shift in mindset is essential to make the necessary progress to increase the speed of implementation of new approaches and enhance innovation on a path to more sustainable agriculture.

An example of an effective change in mindset is the pharmaceutical sector's establishment of the International Council for Harmonisation (ICH) in 1990. With a shared international vision to more efficiently distribute safe medicinal products, three founding regulatory agencies and three funding industry associations initially created a single ‘efficacy‐safety‐manufacturing quality’ paradigm (https://www.ich.org/page/history), with a clear mission of harmonization for better health (https://www.ich.org/page/mission). This resulted in international harmonization while maintaining the possibility of geographical adaptations. A similar framework for agrochemicals is needed. Because new and better agricultural technologies constantly are being developed, flexible frameworks would enable the industry to answer key safety, risk and regulatory questions, and create safe, efficient new technologies and ways to introduce their use.

Both human and environmental safety and risk must be considered in any relevant innovative strategy. The TEA workgroups will identify key questions relevant to both aspects individually, and in combination to create a new integrated agrochemical evaluation paradigm. The goals of this multi‐stakeholder partnership line up with those of the multidisciplinary ‘OneHealth’ concept, which is currently used in the science of food safety and is critical to the implementation of several of the United Nations 2030 Sustainability Objectives (https://learningforhumanity.org/what-makes-us-different/un-2030-agenda-for-sustainable-development/?gclid=EAIaIQobChMIzeC6xJvc9QIV2frICh3AIgU6EAAYAiAAEgJAwfD_BwE).

**Figure 3:**
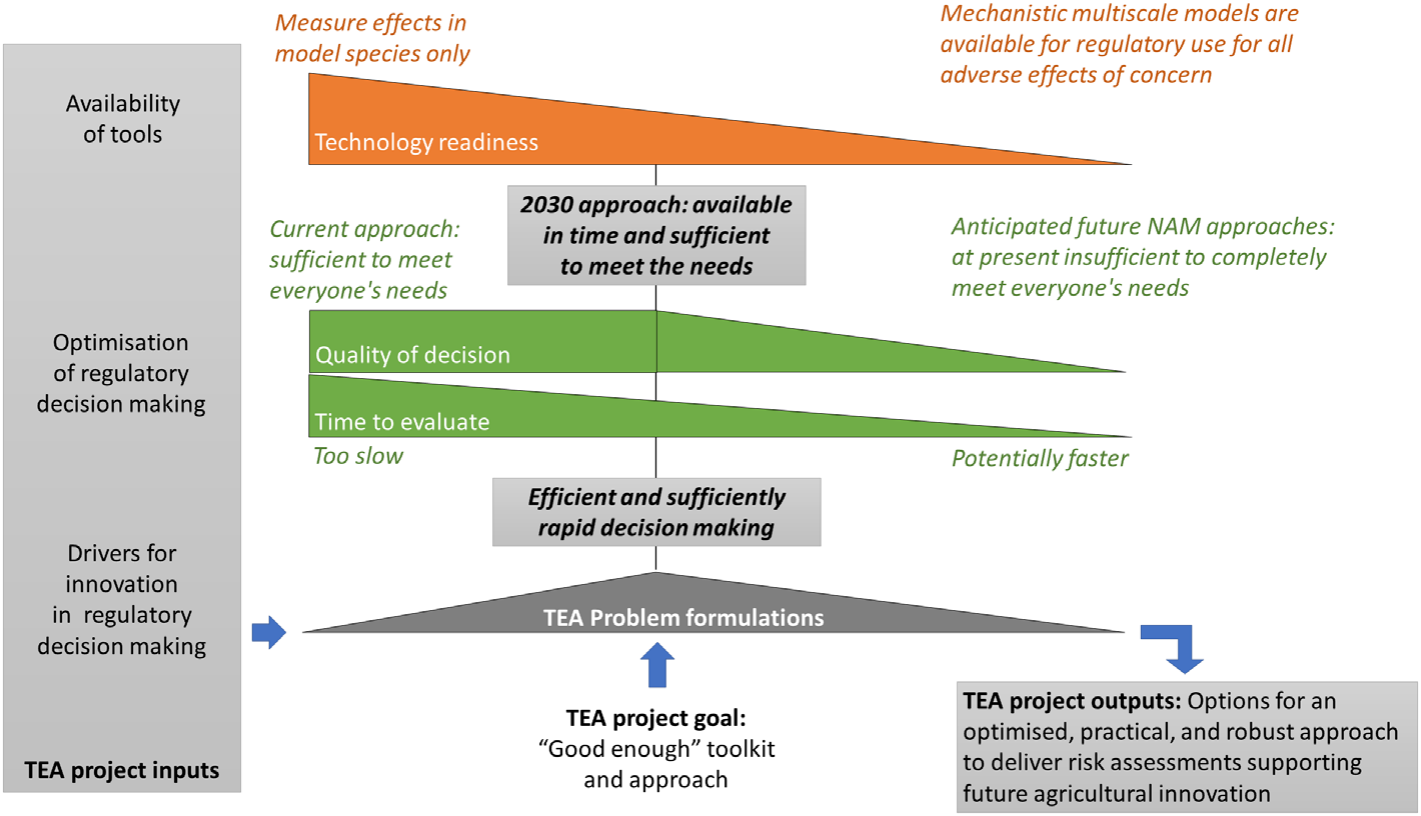
The TEA project will aim to identify what current tools and what additional tool development will be necessary to change the agrochemical testing paradigm quickly and efficiently. The toolkit can be considered as ranging from the current guideline studies in model species where we observe toxicity, to a future state where we have a suite of sufficiently descriptive mechanistic quantitative multiscale models that comprehensively covers the entirety of regulatory endpoints of concern and is rapidly informed by chemical specific data. It is anticipated that mechanistic model‐based approaches will permit more rapid and sustained high quality decision making. However, the current state of the science does not provide similar levels of technological readiness across this full spectrum of tools. Nevertheless, the goal is to make practical improvements to safety assessment and not delay until we create a theoretically perfect system. Therefore, the identification of existing NAMs that both accelerate the speed to decisions, and maintain or improve on the current quality of those decisions, is an important early goal of the project. This output represents the ‘good enough toolkit’, where we can efficiently and confidently implement a sufficiently rapid decision‐making process.

The TEA problem formulation discussions will seek to optimize the regulatory decision‐making process by using available tools, at the same time determining how current practices could evolve to deliver a risk assessment and risk management paradigm that is efficient, practical and as robust as the existing one (Fig. 3). The core of the challenge is that agriculture is evolving quickly and risk assessment and management decisions need to take less, rather than more, time to meet global regulatory and safety needs. Current regulatory risk assessments rely on observing adverse effects in model organisms that act as surrogates for a species of concern. Although this may provide a robust hazard characterization, the process is slow and inefficient and generates large amounts of data that are not always necessary to make critical safety decisions resulting in the unnecessary use of laboratory animals.

Scientific progress has identified many NAMs that involve simple decision trees, read‐across evaluation strategies, quantitative structure–activity relationships (QSARs), adverse outcome pathways (AOPs) and detailed, multiscale mechanistic models of the biology that underlies an adverse event. Many of these tools are based on fit‐for‐purpose modern science that could accelerate risk assessment and risk management decisions, while reducing their inherent uncertainty and increase their robustness. However, often there is a mismatch between the availability and technical readiness of these tools and/or the completeness of coverage of relevant endpoints with the need to incorporate these into safety assessments (Fig. 3).

Significant advances have been made in the development and use of NAMs to support safety assessment, yet improvement is needed to more quickly characterize the many endpoints considered in safety assessments and address all relevant regulatory concerns. The lack of efficiency in qualification of NAMs may prevent the most advanced approaches from being rapidly and generally adopted in the near‐ to mid‐term. However, some existing frameworks would expedite the development and qualification of NAMs if they were widely and regularly implemented.^13^ The TEA project will explore the techno‐regulatory landscape to identify opportunities that are practical, optimized, efficient, and available now or soon, and that will ensure human and environmental safety (Fig. 3).

Improved understanding of chemical exposure will be a key feature in the development of this new paradigm which will better enable the transition to NAMs for safety evaluation. If one knows or can predict human and/or environmental exposures to agrochemicals, then one can inform what hazard information may be necessary for effective safety and risk assessments.^19^ An informed approach will determine which studies should be performed and their design, including dose selection and the potential need for *in vivo* data, and in what species. In addition, exposure information can be used to improve the interpretation of NAMs through improved *in vitro* to *in vivo* extrapolations (IVIVE). Several comprehensive databases, including dietary consumption data, already are available and could be used for such exposure estimates.^19^, ^20^ There also is a wealth of existing industry data that could be made more widely available to develop exposure models and improve predictions. Other publicly available data from, for example, QSARs or read‐across evaluations coupled with mechanistic insights drawn from AOPs, can be used to help select what studies may or may not be necessary.

Global harmonization is a major challenge to the development of a consistent globally applicable data package addressing both human and environmental safety of new active ingredients. This challenge is reflective of differences in evaluation strategies and legislation associated with regional, social, cultural and political history, as well as different pesticide‐use patterns and local environmental considerations. A new framework must have sufficient flexibility to incorporate region‐specific requirements while adhering to best scientific practices. The framework also will need to be adaptable so it can incorporate rapidly evolving scientific methods as NAMs are developed and qualified.

## 5 DISCUSSING THE ROUTE TO IMPLEMENTATION

**Figure 4:**
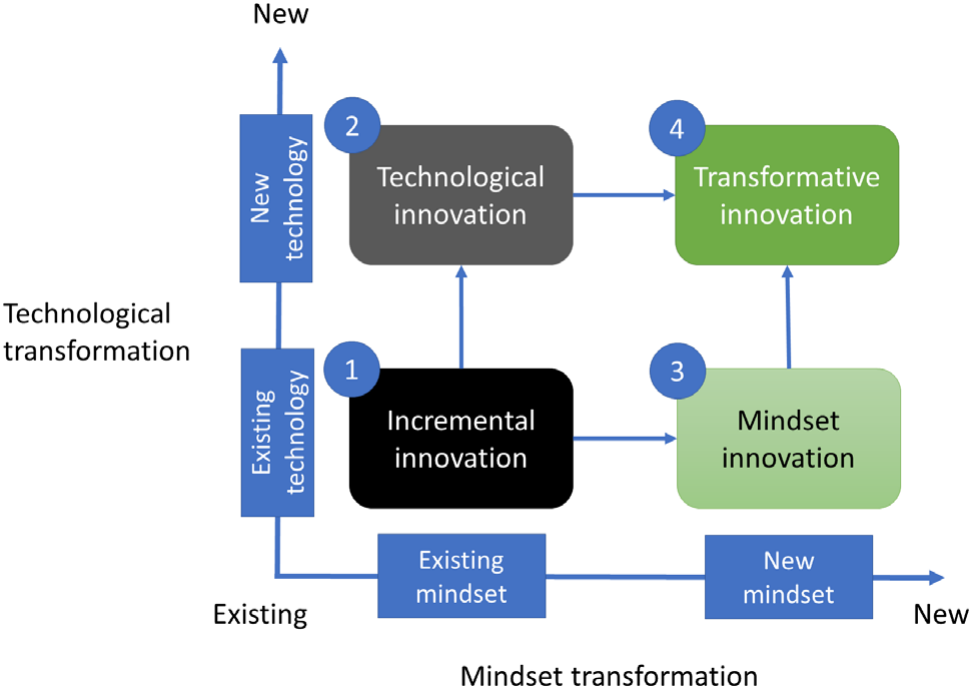
Technical innovations and changes in thinking necessary to transform paradigms. The TEA project will aim to explore routes to implementation of NAMs and whether their continuous improvement and incremental innovation of the existing tools will be sufficient (i). If new technologies can be effectively incorporated into the mindset prevalent in people using the existing paradigm (ii) this will create some burden for acceptance and (re)training. More challenging, but perhaps with the greatest potential for rapid uptake, it may be possible to use existing tools to support safety assessment after a mindset transformation to a new paradigm using already trusted tools (iii). Finally, if new technological tools must be integrated, it will take a mindset change in people who will use the new paradigm built on scientific innovation (Fig. 4, vertical dimension).

Modern agrochemical risk assessment has developed via a path of stable scientific gradualism over decades; however, scientists and human health risk assessors have pushed the boundaries through the development and implementation of alternative tools built on scientific innovation (Fig. 4, vertical dimension), including molecular biology assays, high throughput screening, ‘omics’ technologies, mode and mechanism‐based toxicology, and AOPs. These new data sources have proven that the traditional generalist paradigm of agrochemical risk assessment is no longer adequate to address future problems and concerns without also incorporating conceptual innovation (Fig. 4, horizontal dimension).

Those NAMs that have been implemented have already played an important role in addressing specific data gaps on a case‐by‐case basis. There now is a need to go beyond one‐for‐one replacement by creating a paradigm shift in agrochemical risk assessment to targeted, rapid, relevant and reliable testing that protects humans and the environment, reduces uncertainty, and uses far fewer or no animals. To create such a paradigm shift, it will be necessary to change the mindset of many practitioners in this field and build confidence in major technological innovations to replace the current standard approach (Fig. 4). This will be facilitated by leveraging key aspects of both mindset and technological innovation that build upon incremental scientific achievements to provide guidance on the route towards implementation of new science to optimize agrochemical safety assessment.

### 5.1 Scientific gradualism

The numerous examples of incremental scientific achievements individually represent relatively limited progress in our understanding of a particular endpoint, but collectively contribute significant advancement. For example, for decades, QSAR modelling has been developed and implemented for a more rapid prediction of adverse consequences from exposure to chemicals based on their structure^26^ (https://www.oecd.org/chemicalsafety/risk-assessment/oecd-qsar-toolbox.htm). In more recent years, the AOPWiki, (https://aopwiki.org) has grown to include information on ≈400 adverse outcome pathways (AOPs), >1500 key events, >2200 key event relationships and >700 stressors (metrics accurate as of 21 April 2022 https://aopwiki.org/metrics_summary). Information about individual biological or chemical events associated with an endpoint of interest are collected, shared and evaluated to construct an AOP. AOPs represent a series of measurable changes that can be expected to occur if a perturbation is sufficiently severe to result in an adverse outcome and have been a significant step forward in our understanding of the mechanistic basis of chemically‐induced adverse outcomes, improving regulatory decision‐making and have been used to develop AOP‐specific NAMs.^27^, ^28^ This kind of strategic effort based on a coordinated evaluation of results obtained from multiple NAMs have been shown to perform better than corresponding animal tests, improving human health protection.

### 5.2 Mindset innovation

In order to transform the regulatory perspective, adoption of an outcome‐focused approach to data requirements will be necessary. For example, in the future, for specific use scenarios, regulators could receive targeted data packages that are customized to the risk profile of the proposed product use instead of a long list of studies, many of which end up not being used, to inform a registration or regulatory decision. Sources of uncertainty and variability will need to be addressed to discriminate between a lack of data *versus* incomplete understanding of the risk assessment context, and inherent heterogeneity *versus* the diversity of the data used in an assessment. Understanding the differences between these terms and how they relate to the risk assessment being undertaken will be critical. For example, there should be no need for effects data from rodent studies conducted at 1000 mg kg^−1^ day^−1^ when exposures would be at 1 μg kg^−1^ day^−1^. Uncertainty can be reduced, or eliminated, with more relevant qualitative or quantitative data or analyses. Although variability cannot always be reduced, it can be better characterized to increase understanding of its relevance to the findings of concern.

Mindset innovation (Fig. 4) is arguably the most challenging hurdle to surmount when trying to achieve a transformative vision that drives change. In order to achieve a mindset change it will be important for the TEA project to articulate and understand the many barriers to and drivers of change.^10^, ^22^ Identifying the major barriers to enable change, what drives the establishment of the barriers, potential paths through the barriers, and establishing confidence in a new paradigm will be a key outcome of this project.

A new mindset is needed to accept a transformative change that leads to the implementation of new approaches (Fig. 4).^29^ To transition from exclusively relying upon conventional approaches to including NAMs, regulatory scientists will have to be engaged through an adaptation of design‐thinking. This ‘top‐down’ approach builds on experience with conventional assays required for regulatory decision‐making. Insight from past experience then is incorporated in a process that addresses the issue via phases including proposing options, testing prototypes, learning from successes and failures, and implementing new approaches. The entire process is designed to keep regulatory requirement outcomes at the forefront of the work. In addition, the exploration phase is collaborative and incorporates all stakeholders involved in the problem. This enriches the science, ensures that the solutions meet stakeholder needs, and gives stakeholders confidence in the new technology so that they start using it quickly. This approach then is coupled with other processes that translate, for example, case study findings into applications, which rely on frameworks that incorporate both innovation and acceleration.^29^ Finally, overcoming blocks to change is not strictly a science‐based issue. Regional differences, differences in public opinion, and intransigent positions all will need to be identified and solutions found (Fig. 4).

### 5.3 Transformative innovation

The rate of scientific progress in developing and implementing NAMs has been increasing exponentially in recent years and this is reflected in the rapidly growing number of available assays, software platforms and test guidelines. This also is complemented by socio‐political drivers of change and the societal expectation of a ‘toxic‐free’ or ‘zero‐risk’ environment. This is exemplified by the European Chemicals Strategy for Sustainability (CSS).^30^

Addressing the barriers to mindset innovation is necessary but not sufficient to fully realize the transformative innovation of a globally harmonized evaluation strategy that is embraced by all regions. The OECD has been instrumental in establishing harmonized testing methods and creating a forum for international cooperation; however, the pace of global regulatory change has lagged far behind the pace of innovation. In addition, some countries are not members of the OECD, and therefore do not adhere to its Mutual Acceptance of Data (MAD) process. This leads to different national data requirements and the generation of redundant datasets. Science, legislation and policy need to be synchronized to enable agrochemicals to be evaluated with sufficient flexibility to accommodate future scientific and societal advances.

## 6 CONCLUSIONS

Transforming the existing evaluation framework for agrochemicals is necessary and will only happen through merging alternative approaches to animal assays (NAMs) with existing guidelines and successful strategies, based on accepted case studies, that have been used to evaluate the human and environmental safety of agrochemicals. As with any change process, there will be hurdles; but excellent momentum has been built by existing multi‐stakeholder collaborative efforts that already have set the stage for this transformation.^5^ Although the details of the potential challenges have yet to be worked out, the existing multi‐stakeholder and systems‐thinking approaches being used already are producing results through this TEA initiative. It is hoped that this publication will be looked at as a call‐to‐action and the reader will reach out to the corresponding author with input and willingness to participate in this project.

The existing regulatory frameworks for agrochemical evaluations are built partially on a foundation of legislative requirements. Therefore, in jurisdictions with outcome‐based legislation, there may be sufficient flexibility in the existing requirements for regulators to amend their approach and adapt to advances in science; however, there also are many examples where regulatory requirements are prescribed in legislation. In these cases, the paradigm shift in the risk assessment process will take more time and will need to be accompanied by a similar shift in policy or changes in legislation.

## CONFLICT OF INTERESTS

The authors declare that they have no known competing financial interests or personal relationships that could have appeared to influence the work reported in this paper.

## Data Availability

Data sharing not applicable to this article as no datasets were generated or analysed during the current study.
